# Emergency department utilization in patients with hypertrophic cardiomyopathy: a nationwide population-based study

**DOI:** 10.1038/s41598-022-07463-2

**Published:** 2022-03-03

**Authors:** You-Jung Choi, Bongseong Kim, Hyun-Jung Lee, Heesun Lee, Jun-Bean Park, Seung-Pyo Lee, Kyungdo Han, Yong-Jin Kim, Hyung-Kwan Kim

**Affiliations:** 1 Cardiac Diagnostic Test Unit, Section of Cardiovascular Imaging, Division of Cardiology, Cardiovascular Center, Department of Internal Medicine, Seoul National University Hospital, 101 Daehak-ro, Jongno-gu, Seoul, 03080 Korea; 2 Department of Biostatistics, The Catholic University of Korea, Seoul, Korea; 3 Healthcare System Gangnam Center, Seoul National University Hospital, Seoul, Korea; 4 Department of Statistics and Actuarial Science, Soongsil University, Seoul, Republic of Korea

**Keywords:** Cardiology, Health services, Epidemiology, Epidemiology

## Abstract

Despite the increasing burden of hypertrophic cardiomyopathy (HCM) on healthcare resources, data on emergency department (ED) utilization in HCM are lacking. This nationwide population-based study extracted 14,542 HCM patients from the National Health Insurance Service database between 2015–2016, and investigated their ED utilization during a one-year period. The reason for ED utilization was defined as the primary diagnosis upon discharge from EDs. The clinical outcome was defined as hospitalization or all-cause mortality within 90 days after the ED visits. A total of 3209 (22.1%) HCM patients visited EDs within a one-year period (mean age, 66.8 ± 13.8 years; male, 57.4%). The majority (71.1%) of HCM patients who visited the EDs were aged ≥ 60 years. The ED utilization rate was higher in women than in men (26.3% versus 19.7%, *P* < 0.001). Cardiovascular diseases were the most common reason for ED visits (n = 1333, 41.5%). Among HCM patients who visited EDs, 1195 (37.2%) were hospitalized, and 231 (7.2%) died within 90 days. ED visits for cardiovascular disease was associated with a higher 90-day all-cause mortality (adjusted odds ratio, 2.72; 95% confidence interval 1.79–4.12). These findings would serve as a basis for future research to establish medical policies on ED utilization in HCM.

## Introduction

Hypertrophic cardiomyopathy (HCM) is a genetic cardiomyopathy caused by sarcomeric gene mutations with autosomal dominant transmission^1^. Epidemiologic studies have reported that the prevalence of HCM is about 0.2% in the general population, but its prevalence seems to be increasing^2,3^. Over the last decade, HCM has been highlighted as a major cause of sudden cardiac death in young adults^4,5^, and most research on HCM has focused on early diagnosis and risk stratification to prevent sudden cardiac death^6–8^. Thanks to recent progress in the management of HCM, however, the life expectancy of patients with HCM has significantly increased, leading to changes in the main causes of morbidity and mortality in HCM population^9^.

The emergency department (ED) provides an important source of medical care, and the number of visits to the ED has significantly increased, disproportionately exceeding the growth of the general population^10^. Considering the expanded lifespan of HCM patients, the utilization of EDs is expected to progressively increase, which will inevitably be accompanied by a dramatic increase in the cost of healthcare^10^. Given this situation, understanding the utilization of EDs in this population is of practical value from the perspective of medical resource allocation.

This study aimed to investigate the characteristics of ED utilization in HCM patients in the era of contemporary management and explore the outcomes of those who were discharged from EDs.

## Results

### Baseline characteristics

A total of 3209 patients visited EDs within the specified one-year period (average age, 66.8 ± 13.8 years; male, 57.4%). The majority of HCM patients who visited the EDs were ≥ 60 years old (n = 2281, 71.1%). This was especially true for women, in whom the proportion of patients aged ≥ 60 years approached 84.5% (n = 1154) (Supplementary Fig. 1).

Compared to those who did not visit EDs, HCM patients who visited EDs had a higher proportion with low-income status, and had a higher prevalence of comorbidities such as hypertension, diabetes mellitus, heart failure, stroke/transient ischemic attack (TIA)/thromboembolism, myocardial infarction, atrial fibrillation/flutter, chronic obstructive pulmonary disease, and chronic kidney disease (Table 1). Among HCM patients who visited EDs, 836 of 855 (97.8%) patients with a history of stroke/TIA/thromboembolism were on oral anticoagulants, whereas among those who did not visit EDs, 1316 of 1629 (80.8%) were taking oral anticoagulants. A history of implantable cardioverter-defibrillator (ICD) implantation was more frequently reported in HCM patients who visited EDs than those who did not (n = 75, 2.34% versus. n = 210, 1.85%, *P* = 0.047). The duration of disease in those who visited EDs was significantly shorter than that in those who did not (61.3 ± 65.6 months versus 70.0 ± 64.4 months, *P* < 0.001).

**Table 1:** Baseline characteristics of patients with hypertrophic cardiomyopathy.

Variables	ED visit (-) N = 11,333	ED visits ( +) N = 3209	*P* value
Age, years	62.3 ± 13.2	66.8 ± 13.8	< 0.001
Men, n (%)	7511 (66.3)	1843 (57.4)	< 0.001
Low-income, ^a^ n (%)	2009 (17.7)	699 (21.8)	0.002
**Comorbidities, n (%)**			
Hypertension	6771 (59.8)	2146 (66.9)	< 0.001
Diabetes mellitus	1952 (17.2)	737 (23.0)	< 0.001
Dyslipidemia	5680 (50.1)	1617 (50.4)	0.131
Heart failure	3782 (33.4)	1530 (47.7)	< 0.001
Stroke/TIA/thromboembolism	1629 (14.4)	855 (26.6)	< 0.001
Myocardial infarction	570 (5.03)	308 (9.6)	< 0.001
Atrial fibrillation/flutter	1817 (16.0)	842 (26.2)	< 0.001
COPD	1129 (10.0)	523 (16.3)	< 0.001
Chronic kidney disease	887 (7.8)	460 (14.3)	< 0.001
ICD implanted, n (%)	210 (1.9)	75 (2.3)	0.047
Disease duration of HCM, months	70.0 ± 64.4	61.3 ± 65.6	< 0.001

*COPD* chronic obstructive pulmonary disease, *ED* emergency department, *HCM* hypertrophic cardiomyopathy, *ICD* implantable cardioverter-defibrillator, *TIA* transient ischemic attack.

^a^Low-income indicated the lowest quartile (25%) of income level.

### EDs utilization

**Figure 1:**
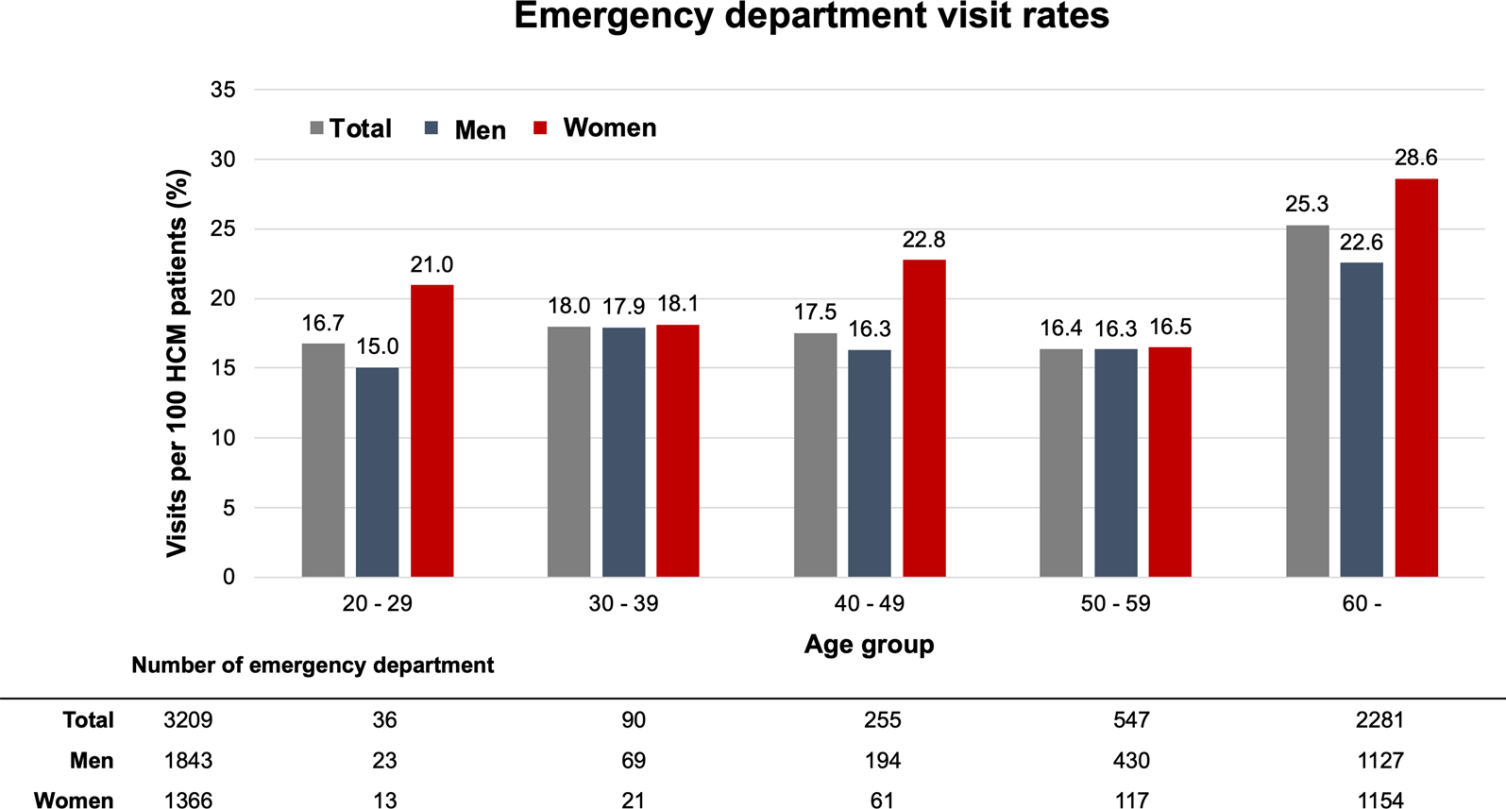
Annual emergency department visit rates in patients with hypertrophic cardiomyopathy (HCM).

During the specified one-year period, 8.8 HCM patients per day utilized EDs. Annual ED visit rate was 22.1% (3209 out of 14,542); 16.8% (928 out of 5513) by HCM patients aged < 60 years, and 25.3% (2281 out of 9029) of those aged ≥ 60 years. Overall, the ED visit rate was significantly higher in women than in men (1366 out of 5188, 26.3% versus 1843 out of 9354, 19.7%, *P* < 0.001). Higher ED visit rates in women were consistent in almost all age groups (Fig. 1).

The average number of ED utilization per year was 1.7 ± 1.8, and most of these patients (n = 2061, 64.2%) utilized EDs only once during a 1-year follow-up period. There were 107 (3.33%) HCM patients who visited EDs more than four times a year. HCM patients in their 20 s had the lowest frequency of ED utilization (1.4 ± 0.6), while those in their 30 s had the highest frequency (2.0 ± 2.6), followed by those in their 40 s (1.8 ± 4.3), 50 s (1.6 ± 1.3), and those 60 or over (1.7 ± 1.4) without a statistical difference between them. (Supplementary Table 2).

**Figure 2:**
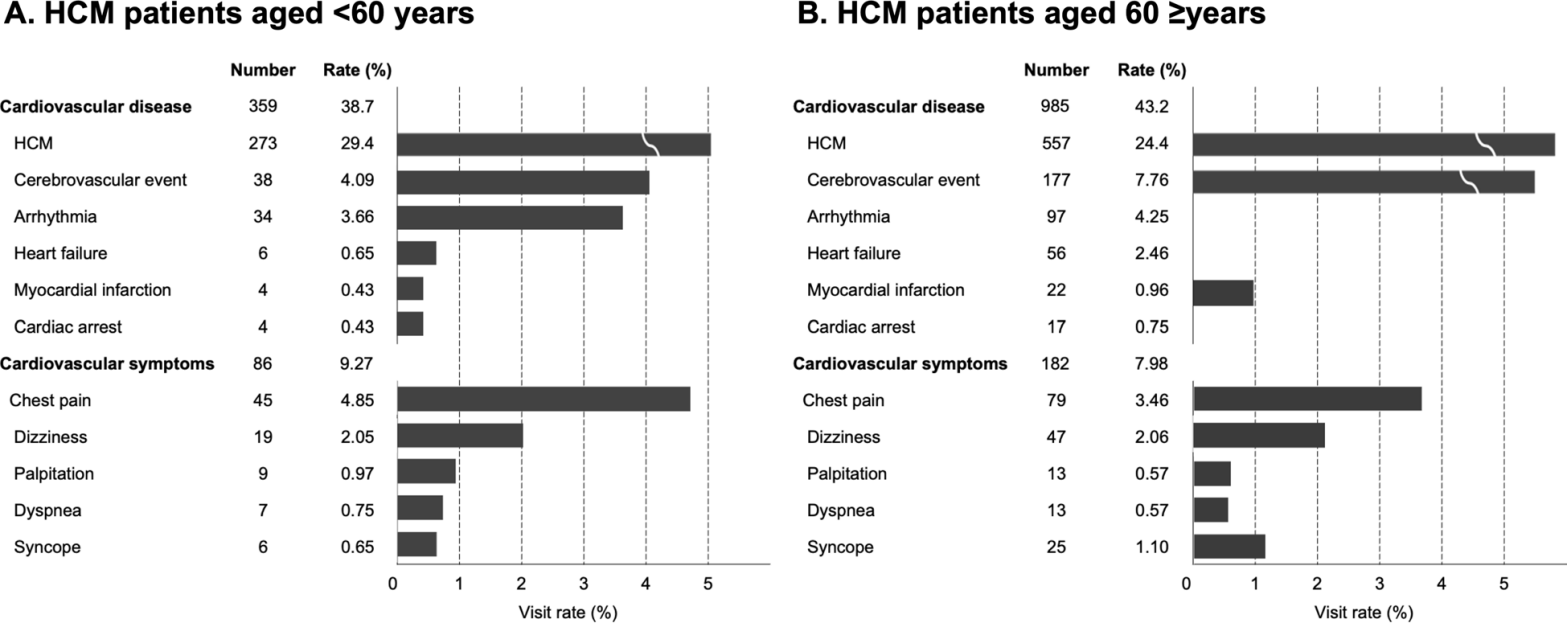
Cardiovascular disease and cardiovascular symptoms according to the primary diagnosis at discharge from the emergency department. Hypertrophic cardiomyopathy (HCM) patients (**A**) aged < 60 years and (**B**) aged ≥ 60 years.

Among the disease categories according to the primary diagnosis upon discharge from the EDs, cardiovascular disease was the most frequently reported primary reason for ED utilization (n = 1344, 41.9%) irrespective of age category (n = 359, 38.7% for patients aged < 60 years; n = 985, 43.2% for patients aged ≥ 60 years) (Supplementary Table 3). Within the category of the cardiovascular disease, HCM was the most common single diagnosis in patients aged both < 60 years (n = 273, 29.4%) and ≥ 60 years (n = 557, 24.4%), followed by cerebrovascular event (n = 38, 4.1% and n = 177, 7.8%, respectively), arrhythmia (n = 34, 3.7% and n = 97, 4.3%, respectively), and heart failure (n = 6, 0.7% and n = 56, 2.5%, respectively) (Fig. 2). Atrial fibrillation/flutter as an individual diagnosis accounted for half of arrhythmias in patients aged < 60 years (n = 17, 50.0%) and over half in those aged ≥ 60 years (n = 60, 61.9%). Detailed diagnoses for cerebrovascular events and arrhythmias based on *International Classification of Disease, Tenth Revision, Clinical Modification* (ICD-10-CM) codes are described in Supplementary Table 4.

Undiagnosed cardiovascular symptoms including chest pain, dizziness, palpitations, dyspnea, and syncope, recorded as the presenting complaint at discharge, accounted for 8.4% (n = 268) of the primary diagnosis of all the ED visits; 9.3% (n = 86) in those aged < 60 years, and 8.0% (n = 182) in those aged ≥ 60 years (Supplementary Table 3). Among undiagnosed cardiovascular symptoms, chest pain was the most common in both patients aged < 60 years (n = 45, 4.9%), and those aged ≥ 60 years (n = 79, 3.5%), followed by dizziness (n = 19, 2.1% and n = 47, 2.1%, respectively). The third most common undiagnosed cardiovascular symptom in patients aged < 60 years was palpitations (n = 9, 1.0%), while it was syncope in those aged ≥ 60 years (n = 25, 1.1%) (Fig. 2).

### Short-term outcomes after ED utilization

Of 3209 HCM patients who visited EDs, 1195 (37.2%) were hospitalized, and 231 (7.2%) died within 90 days of ED discharge. Of the 231 deaths, cardiovascular causes accounted for 123 (53.2%). Among patients aged 60 years and above, 105 of 207 (50.7%) died from cardiovascular causes, whereas only 30 (14.5%) died from cancer (Supplementary Table 5). HCM patients aged ≥ 60 years were associated with a higher 90-day hospitalization (adjusted odds ratio [OR] 1.78, 95% confidence interval [CI] 1.48–2.14) and all-cause mortality (adjusted OR 3.30, 95% CI 2.10–5.18). Sex difference in mortality was also noted; men had higher all-cause mortality than women within 90 days after ED discharge (adjusted OR 1.35, 95% CI 1.01–1.80). Among the pre-specified subgroups, those with a previous history of stroke/TIA/thromboembolism and atrial fibrillation/flutter, and ICD implantation were significantly associated with a higher 90-day hospitalization rate (Table 2). In addition, HCM patients who visited EDs for cardiovascular disease showed a significant association with a higher 90-day all-cause mortality (adjusted OR 2.72, 95% CI 1.79–4.12).

**Table 2:** Short-term outcomes within 90 days after discharge from emergency department.

Categories	Event number (%)	Unadjusted odds ratio (95% CI)	Age and sex adjusted odds ratio (95% CI)	Multivariable adjusted odds ratio (95% CI) ^a^
**Hospitalization**				
**Age**				
< 60 years old	240 (25.9)	1 (reference)	1 (reference)	1 (reference)
≥ 60 years old	955 (41.9)	2.065 (1.744–2.444)	1.999 (1.680–2.378)	1.781 (1.480–2.144)
**Sex**				
Women	558 (40.9)	1 (reference)	1 (reference)	1 (reference)
Men	637 (34.6)	0.765 (0.662–0.884)	0.932 (0.800–1.086)	0.936 (0.800–1.095)
**Stroke/TIA/Thromboembolism**				
No	798 (33.9)	1 (reference)	1 (reference)	1 (reference)
Yes	397 (46.4)	1.69 (1.441–1.982)	1.479 (1.256–1.741)	1.412 (1.192–1.673)
**Atrial fibrillation/flutter**				
No	826 (34.9)	1 (reference)	1 (reference)	1 (reference)
Yes	369 (43.8)	1.455 (1.240–1.708)	1.327 (1.128–1.562)	1.190 (1.005–1.410)
**ICD implantation**				
No	1159 (37.0)	1 (reference)	1 (reference)	1 (reference)
Yes	36 (48.0)	1.573 (0.994–2.489)	1.974 (1.235–3.155)	1.779 (1.105–2.864)
**Reasons for ED utilization**				
Non-CV disease	1133 (37.4)	1 (reference)	1 (reference)	1 (reference)
CV disease	62 (35.0)	0.904 (0.658–1.241)	0.784 (0.568–1.082)	0.724 (0.522–1.004)
**All-cause mortality**				
**Age**				
< 60 years old	24 (2.6)	1 (reference)	1 (reference)	1 (reference)
≥ 60 years old	207 (9.1)	3.759 (2.446–5.778)	3.805 (2.459–5.886)	3.295 (2.098–5.176)
**Sex**				
Women	108 (7.9)	1 (reference)	1 (reference)	1 (reference)
Men	123 (6.7)	0.833 (0.637–1.090)	1.338 (1.007–1.779)	1.346 (1.005–1.803)
**Stroke/TIA/Thromboembolism**				
No	158 (6.7)	1 (reference)	1 (reference)	1 (reference)
Yes	73 (8.5)	1.298 (0.972–1.733)	0.961 (0.715–1.294)	0.944 (0.695–1.283)
**Atrial fibrillation/flutter**				
No	157 (6.6)	1 (reference)	1 (reference)	1 (reference)
Yes	74 (8.8)	1.357 (1.017–1.810)	1.152 (0.858–1.545)	1.045 (0.770–1.418)
**ICD implantation**				
No	226 (7.2)	1 (reference)	1 (reference)	1 (reference)
Yes	5 (6.7)	0.919 (0.367–2.300)	1.411 (0.550–3.620)	1.292 (0.498–3.356)
**Reasons for ED utilization**				
Non-CV disease	196 (6.5)	1 (reference)	1 (reference)	1 (reference)
CV disease	35 (19.8)	3.566 (2.397–5.306)	2.935 (1.949–4.418)	2.717 (1.790–4.124)

*CI* confidence interval, *CV* cardiovascular, *ED* emergency department, *TIA* transient ischemic attack.

^a^Adjustment for age, sex, low-income, hypertension, diabetes mellitus, dyslipidemia, heart failure, stroke/TIA/thromboembolism, myocardial infarction, atrial fibrillation/flutter, chronic obstructive pulmonary disease, chronic kidney disease, and implantable cardioverter-defibrillator implantation.

## Discussion

This study provides some important epidemiological findings in patients with HCM in terms of ED utilization. The annual ED visit rate of HCM patients was 22.1%, in whom elderly HCM patients aged ≥ 60 years accounted for more than two-thirds of total ED utilization. Among the HCM patients who visited EDs, 37.2% were hospitalized, and 7.2% died within 90 days after discharge from EDs. As expected, elderly patients aged ≥ 60 years were associated with a higher 90-day hospitalization rate and all-cause mortality. Of note, the proportion of ED visits was significantly and consistently higher in women than in men at almost all age groups, and men were associated with higher all-cause mortality. The most common reasons for ED utilization in HCM patients were the manifestations of cardiovascular diseases. More importantly, patients visiting EDs for cardiovascular disease had a higher all-cause mortality rate within 90 days after discharge from EDs compared to those for non-cardiovascular disease.

ED utilization is progressively increasing around the world, and thus overcrowding in EDs is now a global issue^11,12^. In addition to the increase in the volume of ED visits exceeding the growth rate of the population, medical costs per ED visit have also significantly increased^13^. Cardiovascular diseases were previously reported to be one of the most common reasons for ED utilization^11^. This study, which included HCM patients, also demonstrated that the most common disease category for ED utilization was cardiovascular disease. Given the increased longevity of HCM patients, this observation is not surprising, and we can also expect that cardiovascular events other than sudden cardiac death will be increasing in HCM patients as the main reason for ED utilization in the upcoming era. More importantly, this study noted that ED utilization due to cardiovascular diseases was associated with an increase in the 90-day all-cause mortality after ED discharge, and therefore, further studies for improving the prognosis in these patients are warranted.

The diagnosis of HCM is increasing in the elderly population, possibly owing to advanced imaging modalities and enhanced physician awareness^3,14^. Many observational cohort studies have found that most patients with HCM can enjoy a normal life expectancy without functional disability related to disease-associated clinical events nor the necessity for therapeutic intervention^14–16^. On the contrary, the current study has two different revelations; i) older HCM patients visiting EDs and ii) a higher frequency of comorbidities. It has been reported that elderly HCM patients carry multiple chronic comorbidities, which inevitably lead to frequent utilization of EDs^17^. In fact, the present study demonstrated that a quarter of HCM patients aged ≥ 60 years visited EDs at least once a year, and they accounted for more than 70% of the total HCM population who utilized EDs. Particularly, we found that HCM patients who visited EDs were not only older but had a higher prevalence of comorbidities such as hypertension, diabetes mellitus, heart failure, stroke/TIA/thromboembolism, myocardial infarction, atrial fibrillation/flutter, chronic obstructive pulmonary disease, and chronic kidney disease compared to those who did not visit EDs. When these facts are taken together, it is not surprising that HCM patients who visited EDs, especially elderly patients, had a higher hospitalization rate and all-cause mortality within 90 days after ED visits. Therefore, given the increasing frequency of HCM diagnosis and frequent ED utilization in the elderly HCM population, it is the right moment to discuss healthcare strategy for this population, which can be helpful for providing enhanced medical care and reducing the frequency of ED utilization, and ultimately improving their prognosis^18^.

Whereas the prevalence of HCM is higher in men in the general population, women with HCM tend to be older and have more symptoms with greater impairment in exercise performance and more frequent sudden cardiac death events compared to men with HCM^6,19,20^. We observed in the present study that the annual ED visit rate was significantly higher in women than in men. Moreover, the proportion of ED visits was consistently higher in women than in men at almost all age groups. These findings are in line with those of previous studies reporting the presence of sex differences in ED utilization^21,22^, implying the vulnerability of female HCM patients^23^. However, all-cause mortality within 90 days after ED visits was significantly higher in male HCM patients. This finding contrasts with previous observations showing no difference in HCM-related mortality between men and women in the general HCM population^23,24^. Sex differences in all-cause mortality after discharge from ED might be attributed to a variety of factors, such as disease severity and the presence of comorbidities^25,26^. Specifically, compared to the previous study^23^, HCM patients enrolled here were older and thus had a higher incidence of comorbidities such as hypertension, heart failure, and coronary artery disease like myocardial infarction, which could lead to an increase in non-HCM related mortality. Therefore, men with HCM (esp. those aged ≥ 60 years) who visited EDs with several comorbidities need to be regarded as those at increased risk and should be monitored closely. Further studies are required to investigate factors that are associated with sex differences in ED-related prognosis.

Ischemic stroke and atrial fibrillation/flutter are well-known complications of HCM^27^. In the current study, cerebrovascular events and arrhythmias were the second and third most common primary diagnoses upon discharge from EDs. Particularly, stroke is a devastating event leading to death or severe disability and has been reported to occur more frequently in HCM patients with atrial fibrillation/flutter than in those without^27,28^. In this respect, every effort should be made in HCM patients to detect atrial fibrillation/flutter early, and establish effective anticoagulation therapy in the outpatient clinic for preventing embolic events; this will lead to reduced stroke rates and less frequent ED utilization in HCM patients, finally helping to reduce medical costs and improve prognosis^29,30^.

Though once regarded as a rare, intractable condition with a grave prognosis and limited management options, HCM is now recognized as a worldwide, relatively common, and treatable form of genetic cardiomyopathy^31^. As HCM affects approximately 20 million people worldwide, the volume of patients presenting to the ED for management of acute problems is also expected to increase^4^. To the best of our knowledge, this is the first study to investigate and characterize the pattern of ED utilization in HCM patients. We expect our findings to serve as a basis for future research on high-risk HCM patients for ED utilization to better establish medical resource allocation policies. Moreover, future studies must focus on what preventive strategies are needed to reduce ED utilization of elderly HCM subjects with the better clinical management of comorbidities.

Several limitations need to be addressed. First, the current nationwide cohort study only included patients who had a record of HCM diagnosis in the NHIS database between 2015 and 2016 and reviewed their one-year history of ED utilization. Hence, data on annual trends, long-term and subsequent prognosis were not available. Second, the inherent limitation of the NHIS claims database did not allow for detailed data on special investigations such as echocardiography or electrocardiography, as well as information on a history of ICD shock and the location and circumstances of death. Thus, the minor possibility of misdiagnosis cannot be completely excluded. However, the diagnosis of HCM was formally verified with reviews by independent medical experts and healthcare professionals according to a governmental act on national health insurance. Besides, we also previously performed validation of the diagnosis^27^. Finally, due to the nature of claims data on ED utilization, only the main diagnosis recorded was used for analysis.

## Conclusion

In conclusion, we can state that a quarter of HCM patients utilized EDs at least once a year after HCM diagnosis. Cardiovascular diseases such as cerebrovascular events, arrhythmias, and heart failure were the most common reasons for ED utilization. Thus, given the expanded life expectancy and improved preventive strategies for sudden cardiac death associated with HCM, greater emphasis should be placed on the continued surveillance of risk factors and their modification to prevent complications in the contemporary HCM population.

## Methods

### Study design and database

This study is a nationwide population-based epidemiological study using the National Health Insurance Services (NHIS) database. The NHIS is an obligatory universal health insurance program managed by the Korean government since 1989, and offers comprehensive medical care covering 97.2% of the entire Korean population. The NHIS database is based on individual medical bill expenses claimed by NHIS, and includes each patient’s demographics, diagnoses, healthcare utilization, and prescription data^32^.

The Institutional Review Board of Seoul National University Hospital approved the study protocol (E-2004-016-1114) and waived informed consent because the analysis used fully anonymized information. The study conformed to the ethical guidelines of the Declaration of Helsinki.

### Study population

From the NHIS database, we extracted data on HCM patients between January 2015 and December 2016 by using the information on (1) claims for the diagnostic codes (ICD-10-CM) of I42.1 or I42.2 with at least one admission or outpatient clinic visit, and (2) registration in the *rare intractable disease* (RID) program. To investigate adult patients with HCM, we excluded patients aged 18 years or below.

The government-implemented RID program is a welfare policy extending health insurance coverage of up to 90% of medical costs for patients with RID (including HCM since 2008). Therefore, consistent with the act established by the Ministry of Health and Welfare, RID registration is tightly controlled by verification with clinical and imaging evidence, attending physician’s certification, and independent reviews by medical experts and health insurance professionals. This definition of HCM was validated with 1110 patients with HCM by showing that sensitivity and specificity of HCM diagnosis in combination with RID code was 91.5% and 100%, respectively, and the accuracy was 92.6%^6^.

### Measurements

We obtained data on the utilization of ED during a one-year period in patients with HCM. Individuals who visited EDs were defined by codes for emergency medical management charges from the NHIS database (AC101, AC103, AC105, V1100, V1200, V1210, V1300, V1310, V1320, and V1400).

The comorbidities were defined as one or more relevant diagnostic codes during hospitalization, or at least two records of diagnostic codes in outpatient clinics prior to study enrollment. A low income indicated that the patient belonged to the lowest quartile (25%) of income level. Disease duration of HCM was defined as the time from the date of the first HCM diagnosis recorded in the NHIS database to the date of study enrollment. Specific definitions for comorbidities based on ICD-10-CM codes are described in more detail in Supplementary Table 1. These definitions have been previously validated^3,32–34^.

### Outcomes

The reasons for ED utilization were defined from the primary diagnoses recorded upon discharge from the EDs, and were categorized into cardiovascular disease, gastrointestinal disease, respiratory disease, trauma, etc. Meanwhile, non-specific complaints, such as chest pain (that was not typical of angina), dizziness, palpitations, shortness of breath, and syncope, were classified as ‘undiagnosed cardiovascular symptoms.’ We investigated the clinical outcomes of interest as follows: (1) hospitalization and (2) all-cause mortality, both within 90 days after ED visit. Deaths and their causes were confirmed using the National Death Registration Records of Korea. Death from cardiovascular disease was defined based on the ICD-10-CM code of I00-I99, and death from cancer was determined based on the ICD-10-CM code of C00-D49.

### Statistical analysis

For comparison of baseline characteristics, categorical variables (frequencies and percentages) were compared using the χ^2^ test or Fisher’s exact test, while continuous variables (mean ± standard deviation) were compared with the Student’s *t*-test or Wilcoxon’s signed rank-sum test for independent samples. The number of ED visits was presented as mean ± standard deviation, and frequencies and percentages, according to age and sex.

The clinical outcomes were evaluated in the pre-specified subgroups, which were defined according to the baseline characteristics, such as age, sex, a previous history of stroke/TIA/thromboembolism and atrial fibrillation/flutter, ICD implantation, and the reasons for ED utilization (cardiovascular versus non-cardiovascular disease). Multivariate logistic regression analysis was performed to estimate the association between the pre-specified subgroups and outcomes after adjustments for age, sex, low-income, hypertension, diabetes mellitus, dyslipidemia, heart failure, stroke/TIA/thromboembolism, myocardial infarction, atrial fibrillation/flutter, chronic obstructive pulmonary disease, chronic kidney disease, and ICD implantation. The OR was presented with the corresponding 95% CI.

SAS software version 9.4 (SAS, Cary, NC, USA) was used in all statistical analyses, and a *P*-value of < 0.05 was considered statistically significant.

## Supplementary Information

Supplementary Information.

## Data Availability

The datasets generated and/or analysed during the current study are not publicly available due to the data from NHIS that can be analyzed in a designated place.
