# Supplementary material for: Emergency department utilization in patients with hypertrophic cardiomyopathy: a nationwide population-based study
Source: Sci Rep. 2022 Mar 3;12:3534. doi: 10.1038/s41598-022-07463-2 (PMC8894351; doi:10.1038/s41598-022-07463-2)

**Supplementary Information**

**Title:** Emergency Department Utilization in Patients with Hypertrophic Cardiomyopathy: a nationwide population-based study

You-Jung Choi, MD^1^, Bongseong Kim^2^, Hyun-Jung Lee, MD ^1^, Heesun Lee, MD^1, 3^, Jun-Bean Park, MD, PhD^1^, Seung-Pyo Lee, MD, PhD^1^, Kyungdo Han, PhD^4^, Yong-Jin Kim, MD, PhD^1^, Hyung-Kwan Kim, MD, PhD^1^

^1^Department of Internal Medicine, Seoul National University Hospital, Seoul, Korea

^2^Department of Biostatistics, The Catholic University of Korea, Seoul, Korea

^3^Healthcare System Gangnam Center, Seoul National University Hospital, Seoul, Korea

^4^Department of Statistics and Actuarial Science, Soongsil University, Seoul, Republic of Korea

All authors take responsibility for all aspects of the reliability and freedom from bias of the data presented and their discussed interpretation.

**Address for correspondence:**

Hyung-Kwan Kim, MD, PhD

Professor, Director of Cardiac Diagnostic Test Unit, Section of CV Imaging, Division of Cardiology, Department of Internal Medicine, Seoul National University College Hospital

101 Daehak-ro, Jongno-gu, Seoul, 03080, Korea

Fax: +82-2-762-9622, Tel: +82-2-2072-0243

Email: cardiman73@gmail.com or hkkim73@snu.ac.kr

**Supplementary Table 1.** Definitions of covariates

| **Covariates in baseline characteristics** | **ICD-10-CM codes or procedure/device code** | **Additional definitions** |
| --- | --- | --- |
| Hypertension | I10-I13, I15 | Admission ≥ 1 or outpatient clinic ≥ 2 and minimum 1 prescription of anti-hypertensive drug |
| Diabetes mellitus | E11-E14 | Admission ≥ 1 or outpatient clinic ≥ 2 and minimum 1 prescription of anti-diabetic drugs |
| Dyslipidemia | E78 | Admission or outpatient department≥1 |
| Heart failure | I50, I42.0, I11.0, I13.0, I13.2 | Admission ≥ 1 or outpatient clinic ≥ 2 |
| Stroke | I63, I64 | Admission ≥ 1 or outpatient clinic ≥ 2 |
| Transient ischemic attack | G45.8, G45.9 | Admission ≥ 1 or outpatient clinic ≥ 2 |
| Thromboembolism | I74 | Admission ≥ 1 or outpatient clinic ≥ 2 |
| Myocardial infarction | I21, I22 | Admission ≥ 1 or outpatient clinic ≥ 2 |
| Atrial fibrillation/flutter | I48 | Admission ≥ 1 or outpatient clinic ≥ 2 |
| Chronic obstructive pulmonary disease | J43-44 | Admission ≥ 1 or outpatient clinic ≥ 2 |
| Chronic kidney disease | N00-007, N11, I12, N18-19, Q61 | Admission ≥ 1 or outpatient clinic ≥ 2 |
| Implantable cardioverter-defibrillator | (Procedure code and Device code) O0211 + G8301 or 8302, O0212 + G8301 or 8302, O0211 + G8303 |  |

ICD-10-CM, international Classification of Disease, Tenth Revision, Clinical Modification.

**Supplementary Table 2.** Frequency of emergency department utilization in patients with hypertrophic cardiomyopathy during 1 year after diagnosis

| **Frequency** | **Total**  **n = 3,209** | **Sex** | | ***P***  **value** | **Age** | | | | | ***P***  **value** |
| --- | --- | --- | --- | --- | --- | --- | --- | --- | --- | --- |
|  |  | **Men**  **n = 1,843** | **Women**  **n = 1,366** |  | **20s**  **n = 36** | **30s**  **n = 90** | **40s**  **n = 255** | **50s**  **n = 547** | **≥ 60**  **n = 2,281** |  |
| Mean ± SD | 1.67 ± 1.8 | 1.68 ± 2.1 | 1.66 ± 1.3 | 0.726 | 1.44 ± 0.6 | 2.00 ± 2.6 | 1.80 ± 4.3 | 1.56 ± 1.3 | 1.68 ± 1.4 | 0.127 |
| Number of visits, n (%) | |  |  |  |  |  |  |  |  |  |
| 1 | 2,061 (64.2) | 1,119 (65.0) | 863 (63.2) | - | 22 (61.1) | 54 (60.0) | 179 (70.2) | 385 (70.4) | 1,421 (62.3) | - |
| 2 | 703 (21.9) | 391 (21.2) | 312 (22.8) |  | 12 (33.3) | 22 (24.4) | 43 (16.9) | 104 (19.0) | 522 (22.9) |  |
| 3 | 243 (7.6) | 141 (7.7) | 102 (7.5) |  | 2 (5.6) | 6 (6.7) | 18 (7.1) | 26 (4.8) | 191 (8.4) |  |
| 4 | 95 (3.0) | 57 (3.1) | 38 (2.8) |  | 0 (0.0) | 2 (2.2) | 9 (3.5) | 14 (2.6) | 70 (3.1) |  |
| ≥ 5 | 107 (3.3) | 56 (3.0) | 51 (3.7) |  | 0 (0.0) | 6 (6.7) | 6 (2.4) | 18 (3.3) | 77 (3.4) |  |

SD, standard deviation.

**Supplementary Table 3.** Reasons for emergency department utilization in patients with hypertrophic cardiomyopathy

| Categories | Total  n = 3,209 | < 60 years  n = 928 | ≥ 60 years  n = 2,281 |
| --- | --- | --- | --- |
| Cardiovascular disease | 1,344 (41.9) | 359 (38.7) | 985 (43.2) |
| Undiagnosed cardiovascular symptoms | 268 (8.4) | 86 (9.3) | 182 (8.0) |
| Gastrointestinal disease | 364 (11.3) | 83 (8.9) | 281 (12.3) |
| Respiratory disease | 212 (8.5) | 19 (2.1) | 193 (8.5) |
| Trauma or injury | 318 (10.3) | 84 (9.1) | 234 (10.3) |

Data was presented as number (%).

**Supplementary Table 4.** Diagnostic codes for cerebrovascular event and arrhythmia

| **Categories (ICD-10-CM codes)** | **Diagnosis** |
| --- | --- |
| **Cerebrovascular event** |  |
| I60 | Non-traumatic subarachnoid hemorrhage |
| I61 | Non-traumatic intracerebral hemorrhage |
| I62 | Other and unspecified non-traumatic intracranial hemorrhage |
| I63 | Cerebral infarction |
| I64 | Stroke, not specified as hemorrhage or infarction |
| I66 | Occlusion and stenosis of cerebral arteries, not resulting in cerebral infarction |
| G45 | Transient cerebral ischemic attacks and related syndromes |
| I74 | Arterial embolism and thrombosis |
| **Arrhythmia** |  |
| I44 | Atrioventricular and left bundle-branch block |
| I47 | Paroxysmal tachycardia |
| I48 | Atrial fibrillation and flutter |
| I49 | Other cardiac arrhythmias |

ICD-10-CM, international Classification of Disease, Tenth Revision, Clinical Modification

**Supplementary Table 5.** Causes of deaths within 90 days after discharge from the emergency department

| **Categories** | **All-cause mortality** | **CV death** | **Non-CV death** | **Cancer** |
| --- | --- | --- | --- | --- |
| **Total** | 231 (7.2) | 123 (3.8) | 89 (2.8) | 32 (1.0) |
| **Age** |  |  |  |  |
| < 60 years | 24 (2.6) | 18 (1.9) | 4 (0.4) | 2 (0.2) |
| ≥ 60 years | 207 (9.1) | 105 (4.6) | 85 (3.7) | 30 (1.3) |
| **Sex** |  |  |  |  |
| Men | 123 (6.7) | 59 (3.2) | 52 (2.8) | 21 (1.1) |
| Women | 108 (7.9) | 64 (4.7) | 37 (2.7) | 11 (0.8) |
| **Reasons for ED utilization** |  |  |  |  |
| Non-CV disease | 196 (6.5) | 95 (3.1) | 86 (2.8) | 31 (1.0) |
| CV disease | 35 (19.8) | 28 (15.8) | 3 (1.7) | 1 (0.6) |

CV, cardiovascular; ED, emergency department.

**Supplementary Figures 1.** Proportion of patients with hypertrophic cardiomyopathy who visited emergency department according to age and sex


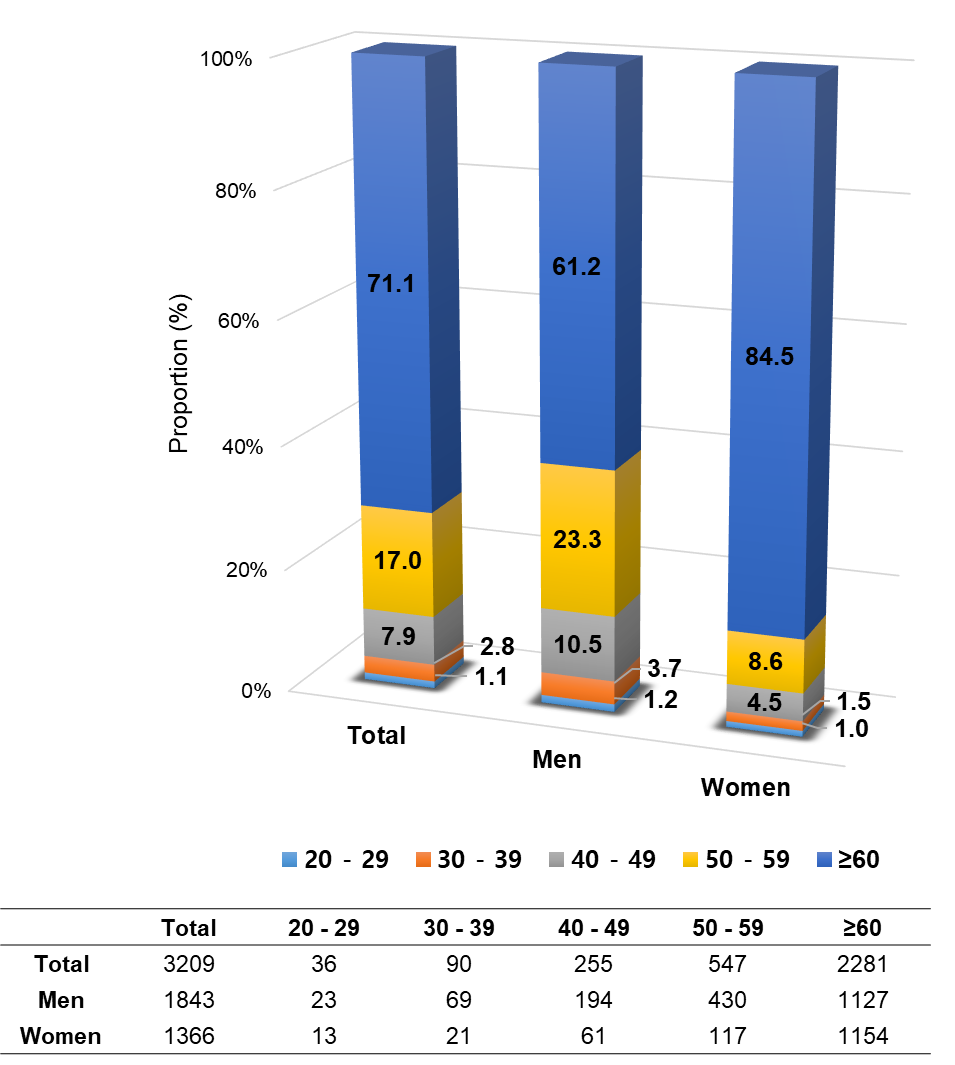

Supplement: Supplementary file 1 — Supplementary Information. [file 41598_2022_7463_MOESM1_ESM.docx]
